# Supplementary material for: Identification of photoperiod-induced specific miRNAs in the adrenal glands of Sunite sheep (Ovis aries)
Source: Front Vet Sci. 2022 Jul 22;9:888207. doi: 10.3389/fvets.2022.888207 (PMC9354845; doi:10.3389/fvets.2022.888207)
Supplement: Supplementary file 1 [file Data_Sheet_1.ZIP › Supplementary file/miRDeep2 file.zip!/miRDeep2 file/Novel_Structure/SP42A2/Novel_218.pdf]

Provisional ID	:	NC_019478.2_37136
Score total	:	1.9
Score for star read(s)	:	-1.3
Score for read counts	:	0
Score for mfe	:	1.6
Score for randfold	:	1.6
Score for cons. seed	:	
Total read count	:	4053
Mature read count	:	4053
Loop read count	:	0
Star read count	:	0

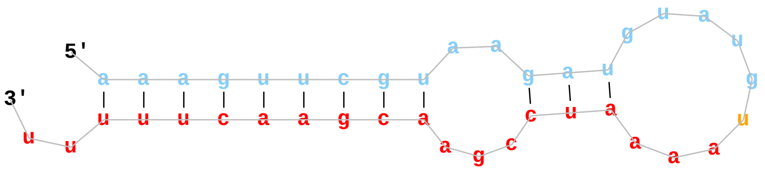

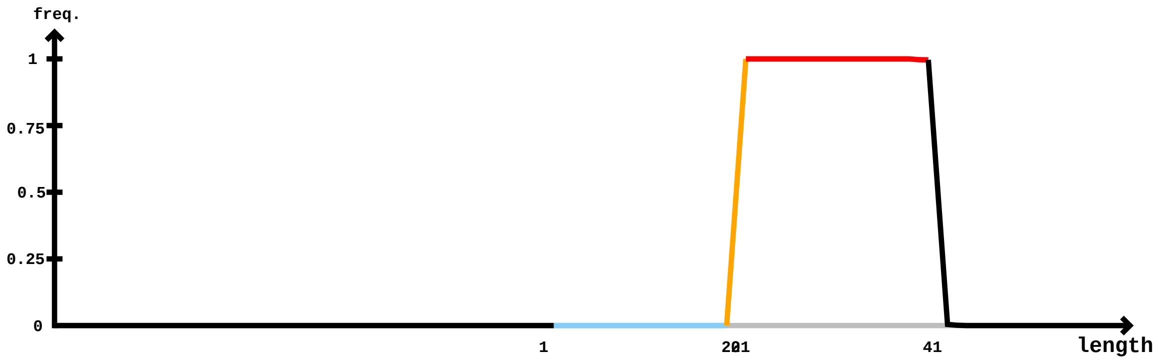

Star Mature

```
5'- gcagaggaaggaaggagggggugucugagaggguauugggcuugggccagaaaguucguaagauguauguaaaauccgaacgaacuuuuugggcccacccaguauuucaa   -3'    exp
    .((((.(..............).))))...(((((((((..((((((((((((((((((..(((.........)))...))))))))))).))))))))))))))))...     reads       mm          sample
    ......................................................................aaaauccgaacgaacuuu......................     11          0           seq
    ......................................................................aaaauccgaacAaacuuu......................     1           1           seq
    ......................................................................aaaaucUgaacgaacuuuu.....................     2           1           seq
    ......................................................................aaaauccAaacgaacuuuu.....................     1           1           seq
    ......................................................................aaaauccgaacgaacuuuuu....................     4015        0           seq
    ......................................................................aaaauccAaacgaacuuuuu....................     5           1           seq
    ......................................................................aaaaucUgaacgaacuuuuugg..................     7           1           seq
    ......................................................................aaaauccAaacgaacuuuuugg..................     1           1           seq
    ......................................................................aaaauccgaacAaacuuuuugg..................     5           1           seq
    ......................................................................aaaauccgaacgaacuuuuuggg.................     5           0           seq
```
